# Efficacy and Safety of Esketamine in Total Hip and Knee Arthroplasty: A Systematic Review and Meta-Analysis of Randomized Controlled Trials

**DOI:** 10.7759/cureus.109496

**Published:** 2026-05-23

**Authors:** Abdulrahman E AlAyyaf, Abdullah M Alharran, Mohammad Al-Mutairi, Waleed B Alazemi, Mohammad A Behbehani, Mezna M Alajmi, Khaled M Altamimi, Abdulmuhsen Alqallaf, Ahmad Y Almohammad

**Affiliations:** 1 Department of Medicine and Surgery, Kuwait Institute for Medical Specialization, Kuwait City, KWT; 2 College of Medicine and Medical Sciences, Arabian Gulf University, Manama, BHR; 3 Faculty of Medicine, University of Jordan, Amman, JOR; 4 Department of Medicine and Surgery, Mubarak Al Kabeer Hospital, Kuwait City, KWT; 5 Department of Orthopedics, Al-Razi Hospital, Kuwait City, KWT

**Keywords:** anxiety, esketamine, meta-analysis, perioperative analgesia, postoperative pain, randomized controlled trials, total hip arthroplasty, total knee arthroplasty

## Abstract

Background: Total hip arthroplasty (THA) and total knee arthroplasty (TKA) are commonly performed procedures, but post-operative recovery is often complicated by significant pain, opioid-related side effects, and psychological distress. Esketamine has been proposed as an adjunct due to its analgesic and antidepressant properties, but its efficacy and safety remain unclear.

Methods: A systematic review and meta-analysis of randomized controlled trials (RCTs) was performed by searching PubMed, Scopus, Web of Science, and Cochrane Central Register of Controlled Trials (CENTRAL) up to July 2025. Dichotomous and continuous outcomes were synthesized using risk ratios (RRs) and standardized or mean differences, each reported with 95% confidence intervals (CIs).

Results: Seven RCTs involving 1,101 patients were included. Esketamine did not significantly reduce pain at rest at 12 or 24 hours. Low-certainty evidence suggested that esketamine may reduce pain during activity at 12 hours (standardized mean difference (SMD): -0.28; 95% CI: -0.45, -0.12; p < 0.001), although this effect was not sustained at 24 hours. Very low-certainty evidence suggested a possible reduction in anxiety scores at post-operative day 7, with no significant effect observed at day 1. No clear effect on depression was observed at any time point. Esketamine was associated with a higher incidence of hallucinations (RR: 4.36; 95% CI (1.48, 12.88); p = 0.01), with no significant differences in delirium, nightmares, or post-operative nausea and vomiting.

Conclusions: Low- to very low-certainty evidence suggests that esketamine may provide modest short-term improvement in activity-related pain and delayed anxiety reduction following THA and TKA. However, findings should be interpreted cautiously given the limited number of studies, clinical heterogeneity, and instability observed in sensitivity analyses. Esketamine was also associated with an increased risk of hallucinations.

## Introduction and background

Total hip arthroplasty (THA) and total knee arthroplasty (TKA) are widely performed surgical procedures and are considered highly effective interventions globally, offering substantial improvements in quality of life for patients with end-stage joint diseases [1]. The aging of the global population is expected to result in a substantial increase in demand for these procedures over the next few decades [2]. Accordingly, several recent advancements have evolved, including robotic-assisted surgery [3,4] and unicompartmental arthroplasty [5], as well as novel MRI-based structural phenotyping, which may aid in predicting disease progression [6]. Although THA and TKA are usually successful, the time after surgery often involves significant complications that can slow healing and decrease patients’ satisfaction. Post-operative pain is frequently severe for patients, leading to opioid use and subsequent side effects, such as nausea, vomiting, and respiratory depression [7]. Additionally, rebound pain, characterized by a rapid and sharp increase in pain, can occur as the effects of regional anesthesia wear off [8]. 

In addition to physical pain, many patients, especially older adults, face a high risk of post-operative delirium (POD), an acute state of confusion associated with deteriorated recovery, more extended hospitalizations, and higher mortality rates [9]. The psychological impact is significant as well, with high rates of post-operative anxiety, depression, and sleep disturbance, all of which can hinder rehabilitation and long-term functional recovery [10,11]. These complex challenges highlight the necessity of innovative, multimodal pain management strategies that can both relieve pain and reduce the risk of related complications. 

Esketamine, which is the S-enantiomer of ketamine, has shown potential in this regard. Esketamine's unique pharmacology, as a potent NMDA receptor antagonist, provides benefits beyond simple analgesia [12,13]. The drug is thought to act by interfering with central sensitization, a critical process in the formation of long-term pain, and exhibits both anti-hyperalgesic and anti-inflammatory properties [14]. Also, increasing evidence indicates that esketamine's fast-acting antidepressant properties make it an appealing choice for tackling the physical and psychological dimensions of recovery after surgery [15].

However, the current evidence regarding esketamine use during THA and TKA is inconsistent, leading to confusing evidence. For instance, while some studies suggest that esketamine can improve hemodynamic stability during anesthesia induction [16,17], its effect on preventing post-operative delirium remains contentious, with randomized controlled trials (RCTs) showing no significant benefit [16-19]. However, the evidence supporting its pain-relieving abilities seems stronger, as studies have shown it reduces rebound pain [18] and provides effective pain relief after surgery when used in patient-controlled intravenous analgesia (PCIA) systems [20]. The effects of esketamine on mood and sleep after surgery are being actively studied, but the findings are inconsistent. 

The varying results could stem from differences in study design, patient groups, and, most importantly, the esketamine dosage and administration schedule. Due to the current conflicting evidence, there is a crucial gap in our understanding of how well and how safely esketamine works for patients undergoing THA or TKA before, during, and after the procedure. Therefore, this systematic review and meta-analysis aims to synthesize the available evidence from RCTs to clarify the role of esketamine in improving clinical outcomes following THA and TKA. 

## Review

Methodology

Protocol Registration

This systematic review was prospectively registered in the International Prospective Register of Systematic Reviews (PROSPERO; CRD420251145872). The study methodology adhered to the Preferred Reporting Items for Systematic Reviews and Meta-Analyses (PRISMA) guidelines [21] and followed recommendations from the Cochrane Handbook for Systematic Reviews of Interventions [22].

Data Sources and Search Strategy

On July 19, 2025, a literature search was systematically conducted by (A.M.A.) across several electronic databases: PubMed, Scopus, Web of Science, and the Cochrane Central Register of Controlled Trials (CENTRAL). The search strategy utilized a combination of keywords related to the procedure and the intervention: “("Esketamine" OR "S-ketamine” OR "S ketamine" OR "Spravato" OR "JNJ-54135419") AND (knee OR hip) AND (arthroplasty OR replac* OR prosthesis OR implant*)”. No restrictions were applied during the search, except in Scopus, where retrieval was limited to titles, abstracts, and keywords. The complete search strategy and database-specific results are presented in Appendix A. In addition, reference lists of included studies were manually screened to enhance completeness and minimize the risk of missing relevant articles.

Eligibility Criteria

RCTs adhering to the following Population, Intervention, Control, and Outcome (PICO) framework were eligible for inclusion: Population (P): Patients undergoing either TKA or THA; Intervention (I): Intravenous (IV) administration of esketamine, irrespective of dosing strategy or concomitant medications. Also, we included either intra-operative esketamine infusion or post-operative PCIA with esketamine; Control (C): Administration of a placebo or no intervention; Outcomes (O): The primary outcomes were post-operative pain severity assessed using validated tools such as the Visual Analog Scale (VAS) or Numerical Rating Scale (NRS). Pain outcomes were analyzed separately according to pain condition (pain at rest and pain during activity) and post-operative timepoint (12 and 24 hours), given the expected clinical variability in pain assessment across included studies. Secondary outcomes comprised intra-operative consumption of opioids and propofol, duration of surgery, post-operative depression, post-operative anxiety, length of hospital stay (LOS), and the incidence of adverse events (post-operative nausea & vomiting (PONV), dizziness, hallucinations, nightmares, and delirium). Hallucinations, delirium, nightmares, and post-operative nausea and vomiting were extracted as reported adverse events from the included trials. Most studies did not utilize standardized psychometric assessment tools specifically for hallucination evaluation. Delirium outcomes were extracted according to the assessment methods reported in the original studies, which varied across trials and included either clinical diagnosis or study-specific assessment tools and post-operative evaluation schedules. 

Studies were excluded if they used quasi-randomized designs, evaluated non-standard routes of esketamine administration (e.g., local infiltration or nebulization), were available only as conference abstracts or proceedings, or were observational, in vitro, or review studies.

Study Selection

The screening and selection of studies were carried out by two independent reviewers (M.M.A. and A.E.A.) using Covidence software (Veritas Health Innovation, Melbourne, Australia). After the automatic removal of duplicates, the remaining unique articles underwent a two-phase screening process. Initially, titles and abstracts were reviewed, followed by a full-text assessment of the potentially eligible studies. Any disagreements between the reviewers were resolved through discussion to reach a consensus.

Data Extraction

A Microsoft Excel spreadsheet (Microsoft Corp., Redmond, WA, USA) was developed for data extraction, which was piloted using the full texts of the included articles. The extraction form was organized into three main sections, which are listed below.

Study characteristics: These included study ID, country, design, total number of patients, treatment protocols, type of surgery, adjuvant analgesia, primary outcome assessment tool, and key inclusion criteria.

Participant baseline characteristics: These comprised age, gender, body mass index (BMI), and American Society of Anesthesiologists (ASA) classification.

Outcome data: These included pain, depression, and anxiety scores at various follow-up intervals, intra-operative opioid consumption, intra-operative propofol consumption, surgery duration, LOS, and the frequency of adverse events.

Two reviewers (A.F.A. and W.B.A.) independently extracted the data. Any discrepancies were resolved through discussion and consultation with a senior author. Dichotomous data were extracted as the number of events and total participants, whereas continuous data were extracted as the mean and standard deviation. We utilized the formulas proposed by Wan et al. [23] to convert data presented as median and interquartile range or range into mean and standard deviation. The online tool WebPlotDigitizer [24] was also used to extract numerical data from graphs.

Risk of Bias and Certainty of Evidence

The methodological quality and risk of bias for each included RCT were assessed using the revised Cochrane Collaboration's Risk of Bias tool (RoB 2) [25]. Two reviewers (M.A.B. and M.M.A.) independently assessed each study across key domains, including selection, performance, reporting, and attrition bias. Any disagreements were resolved through discussion. Furthermore, the overall certainty of evidence was evaluated using the Grading of Recommendations Assessment, Development, and Evaluation (GRADE) framework [26,27]. This approach evaluates domains including risk of bias, inconsistency, indirectness, imprecision, and publication bias. Each factor was carefully assessed, and the rationale for each judgment was documented, with any discrepancies resolved through discussion.

Statistical Analysis

Statistical analyses were conducted using Stata MP version 18 (StataCorp LLC, College Station, TX). Risk ratios (RRs) were used for dichotomous outcomes, while mean differences (MDs) were applied for continuous variables, with results reported alongside 95% confidence intervals (CIs). Standardized MDs (SMDs) were used when outcomes were assessed using different measurement scales. Given the expected clinical heterogeneity across included studies, random-effects models were preferentially applied for pooled analyses, while heterogeneity was assessed using the chi-square (χ²) test and quantified using the I² statistic. Between-study variance was estimated using the DerSimonian-Laird (DL) method. A p-value less than 0.1 for the χ² test or an I² value ≥50% was considered indicative of substantial heterogeneity. Due to the limited number of included studies and outcome events, publication bias testing was not formally performed. Findings were interpreted cautiously in light of the small number of studies, clinical heterogeneity, and sensitivity analysis instability observed for several outcomes [28].

Results

Search Results and Study Selection

**Figure 1:**
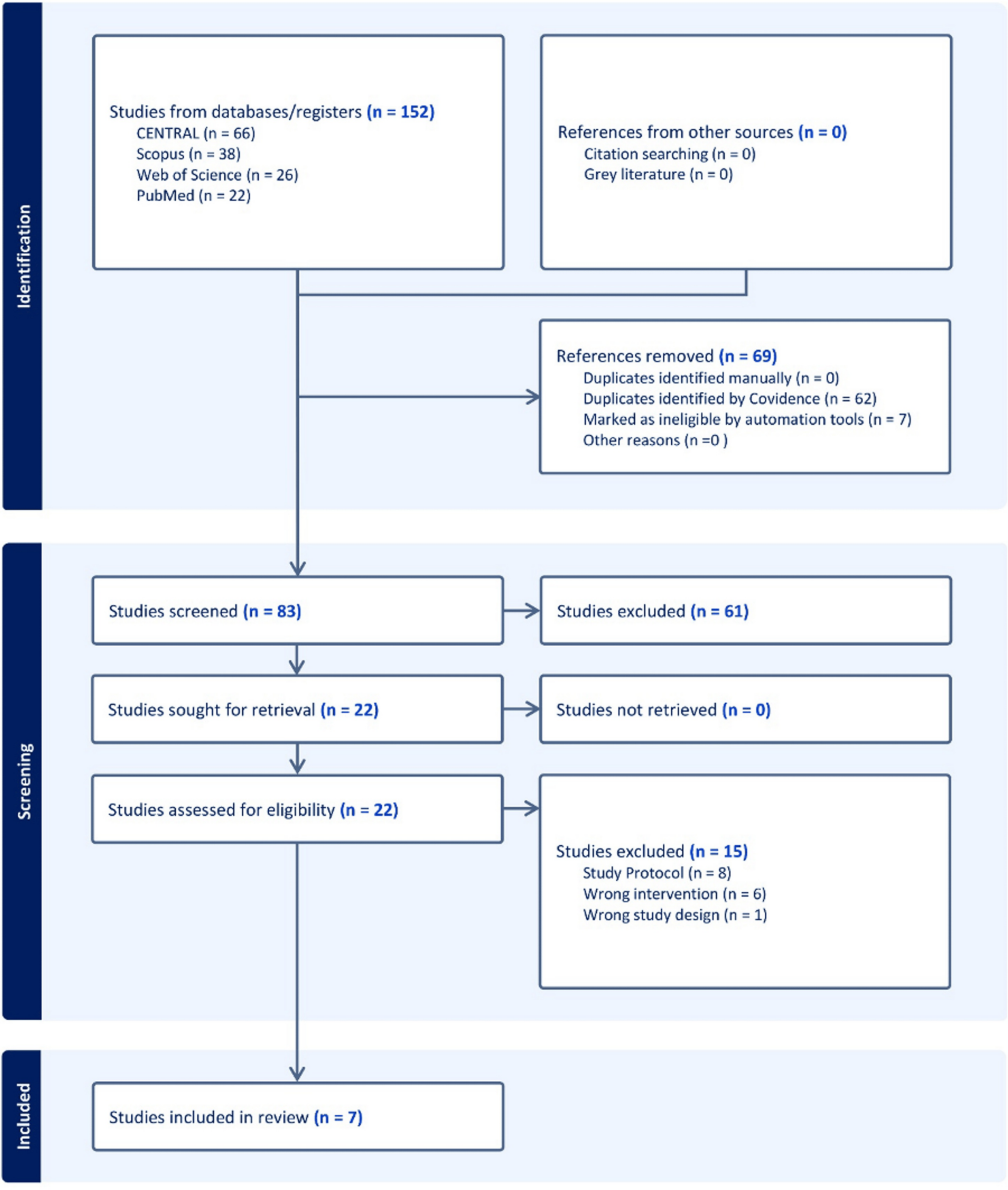
A PRISMA flowchart of the screening process. CENTRAL: Cochrane Controlled Register of Trials

The initial database search identified 152 records. After 69 duplicates were automatically removed, the titles and abstracts of the remaining 83 articles were screened. This led to the exclusion of 61 studies that did not meet the inclusion criteria. Consequently, 22 articles were assessed for eligibility via full-text screening. Of these, 15 studies were excluded for various reasons (Appendix B). Ultimately, seven RCTs (16-20,29,30) were included in the qualitative and quantitative synthesis (Figure 1).

Characteristics of the Included Studies

Seven RCTs and 1,101 patients were included in our pooled analysis [16-20,29,30]. All seven investigated esketamine with various infusion protocols. All included trials were single-blinded or double-blinded, except Qu et al., which was an open-label RCT [20]. Most trials provided adjuvant analgesia, which commonly included PCIA with opioids, regional nerve blocks, and non-steroidal anti-inflammatory drugs. The esketamine group consisted of 554 patients, and the control group consisted of 547 patients. Complete details about the study characteristics and baseline data are available in Tables 1, 2.

**Table 1:** Summary characteristics of included RCTs ASA: American Society of Anesthesiologists; DBP: diastolic blood pressure; FICB: fascia iliaca compartment block; HADS-D: Hospital Anxiety and Depression Scale–Depression subscale; HADS-A: Hospital Anxiety and Depression Scale–Anxiety subscale; HAMA: Hamilton Anxiety Rating Scale; HAMD: Hamilton Depression Rating Scale; HR: heart rate; IV: intravenous; MAP: mean arterial pressure; NA: not applicable; NRS: numerical rating scale; PCIA: patient-controlled intravenous analgesia; POD: post-operative day; RCT: randomized controlled trials; SBP: systolic blood pressure; THA: total hip arthroplasty; TKA: total knee arthroplasty

Study ID	Study Design	Country	Total Participants	Surgery Type	Intra- or Post-operative Administration	Esketamine Details	Control Details	Anesthesia Protocol	Adjuvant Analgesia	Primary Outcome	Pain Assessment Score	Depression Assessment Score	Anxiety Assessment Score	Main Inclusion Criteria	
															Li et al., 2022 [16]	RCT	China	80	TKA	Intra-operative	0.2 mg/kg IV injection during induction	Equal volume of IV normal saline	General anesthesia (etomidate, sufentanil, rocuronium, propofol, remifentanil, sevoflurane)	NA	Hemodynamics (HR, SBP, DBP, MAP) and BIS values during induction	NA	HADS-D	HADS-A	Age 65-85 years, ASA Class II-III, undergoing TKR	
Li et al., 2025 [18]	RCT	China	356	TKA	Intra-operative	Continuous intra-operative IV infusion of S-ketamine (0.30 mg/kg/h)	Continuous IV infusion of 0.9% saline	Spinal anesthesia (ropivacaine), adductor canal block, periarticular drug mixture injections	Celecoxib pre-operative; Post-operative PCIA (sufentanil, butorphanol), acetaminophen-oxycodone, meperidine	Incidence of rebound pain within 12 hours post-surgery	NRS	NA	NA	Age 18-75 years, ASA Class I-III, undergoing TKA	
Ma et al., 2024 [17]	RCT	China	260	THA or TKA	Both Intra and Post-operative	IV esketamine: 0.20 mg/kg at induction, 0.125 mg/kg/h infusion intra-op, 0.5 mg/kg in post-op PCIA	Equivalent volume of IV normal saline at all stages	General anesthesia (etomidate, alfentanil, rocuronium, propofol, remifentanil, desflurane), fascia iliaca/adductor canal block	Post-operative PCIA with hydromorphone; prophylactic palonosetron and flurbiprofen	Incidence of post-operative delirium within the first three days	NRS	NA	NA	Age ≥60 years, ASA Class II-III, undergoing THA or TKA	
Min et al., 2023 [29]	RCT	China	132	THA	Post-operative	Post-operative PCIA with esketamine (2.5 mg/kg)	Post-operative PCIA with sufentanil (2.5 µg/kg)	General anesthesia (midazolam, sufentanil, etomidate, rocuronium, propofol, remifentanil, sevoflurane), fascia iliaca block	NA	Depression rate at seven days post surgery; VAS scores; ambulation time/distance	VAS	HADS-D	HADS-A	Age ≥60 years, ASA Class I-II, undergoing THA	
Qu et al., 2025 [20]	RCT	China	89	THA	Post-operative	Post-operative PCIA with esketamine (1.5 mg/kg) and flurbiprofen axetil	PCIA with sufentanil (2.0 µg/kg) and flurbiprofen axetil	General anesthesia (midazolam, sufentanil, propofol, rocuronium, remifentanil, sevoflurane)	Rescue analgesia with tramadol	Post-operative VAS pain scores at rest and on movement	VAS	SAS-D	SAS-A	Age 18-85 years, ASA Class I-III, undergoing THA	
Wei et al., 2024 [30]	RCT	China	72	THA	Post-operative	Post-operative PCIA with esketamine (100 mg) and dexmedetomidine	Post-operative PCIA with fentanyl (0.7g) and dexmedetomidine	General anesthesia (propofol, remifentanil, cisatracurium), iliac fascia block, lateral femoral cutaneous nerve block	Celecoxib, ice packs, and rescue analgesia with tramadol	Time to first rescue analgesia, dose of rescue analgesics, and post-operative sleep quality	VAS	NA	NA	Age 18-70 years, undergoing selective hip arthroplasty	
Y. Zhao et al. 2025 [19]	RCT	China	112	THA or TKA	Post-operative	Post-operative PCIA with esketamine (0.72 mg/kg) and sufentanil (2 µg/kg)	Post-operative PCIA with sufentanil (2 µg/kg) alone	General anesthesia (etomidate, midazolam, sufentanil, rocuronium, propofol, remifentanil, sevoflurane), femoral and lateral femoral cutaneous nerve block	Rescue analgesia with flurbiprofen axetil	Incidence of post-operative sleep disturbance (PSD) on day 1	NRS	HAMD	HAMA	Age 65-80 years, ASA Class II-III, undergoing THA or TKA	

**Table 2:** Baseline characteristics of the participants ASA: American Society of Anesthesiologists; BMI: body mass index; SD: standard deviation *Data presented as median (interquartile range).

Study ID	Number of Participants in Each Group		Age (Years), Mean (SD)		Gender (Male/Female)		ASA I/II/III/IV		BMI, Mean (SD)		
											Esketamine	Control	Esketamine	Control	Esketamine	Control	Esketamine	Control	Esketamine	Control	
	Li et al., 2022 [16]	40	40	68.8 ± 3.6	69.2 ± 5.4	11/29	9/31	0/36/4/0	0/35/5/0	25.7 ± 2.3	25.6 ± 2.2	
Li et al., 2025 [18]	178	178	66 ± 5.0	66 ± 6.0	42/136	46/132	0/158/20/0	0/154/24/0	26 (24, 29)*	27 (25, 29)*	
Ma et al., 2024 [17]	130	130	69.0 ± 5.4	68.6 ± 4.1	38/92	33/97	0/96/34/0	0/101/29/0	25.5 ± 2.5	25.2 ± 2.2	
Min et al., 2023 [29]	67	65	73.9 ± 6.5	73.9 ± 6.1	32/35	30/35	28/39/0/0	23/42/0/0	22.2 ± 1.3	22.0 ± 1.3	
Qu et al., 2025 [20]	44	45	59.3 ± 14.6	61.3 ± 15.0	19/25	23/22	10/26/8/0	4/32/9/0	22.9 ± 3.1	23.7 ± 3.5	
Wei et al., 2024 [30]	38	34	54 (46, 63)*	51 (39, 61)*	27/11	24/10	0/3/33/2	0/3/28/3	23.5 ± 3.1	23.1 ± 3.6	
Zhao et al., 2025 [19]	57	55	70 ± 4.0	70 ± 5.1	18/39	15/40	0/56/1/0	0/53/2/0	25.5 ± 3.2	25.2 ± 3.3	

Risk of Bias and Certainty of Evidence

**Figure 2:**
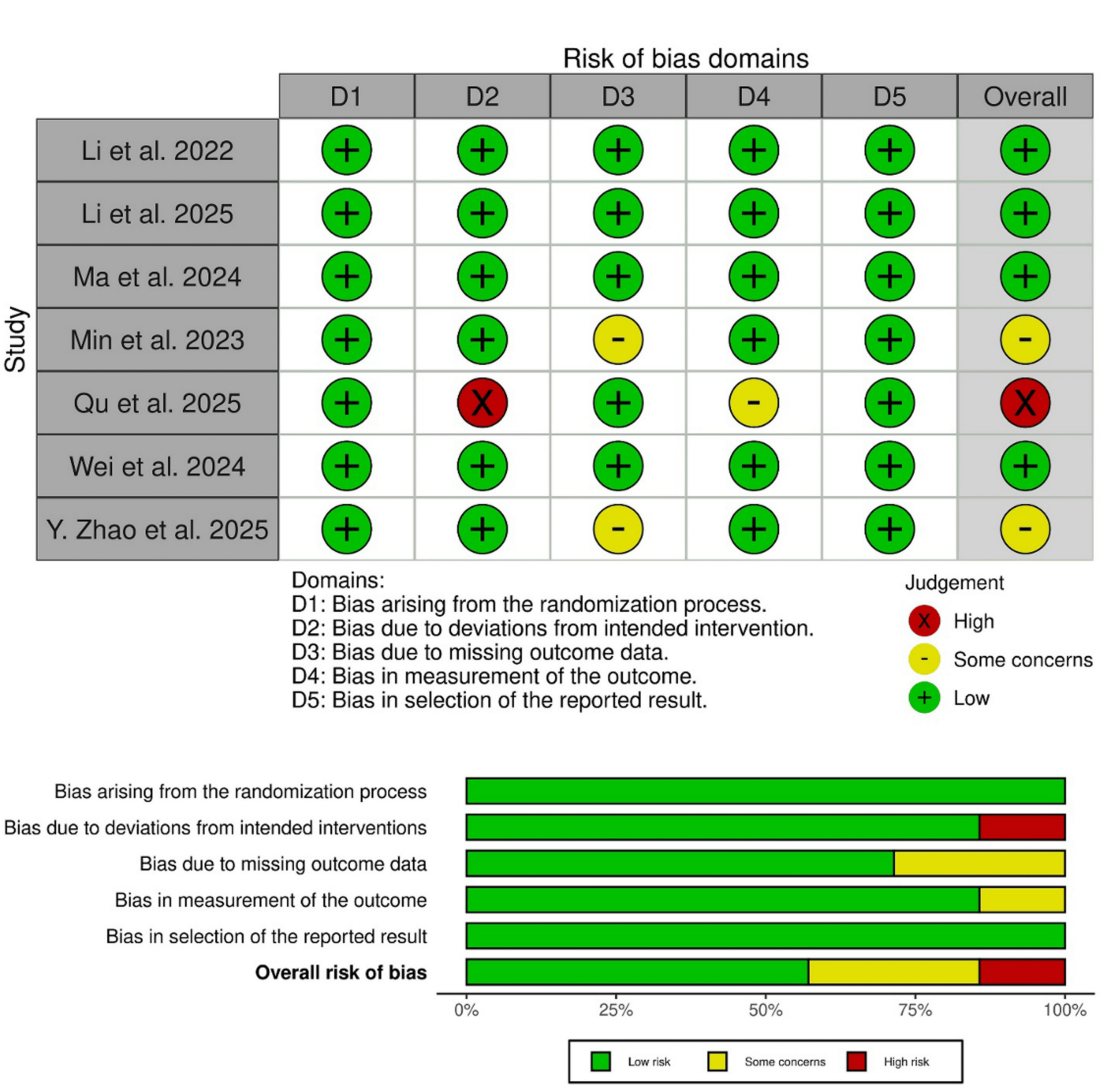
Risk of bias assessment of included randomized controlled trials Risk of bias was assessed using the revised Cochrane Risk of Bias tool for randomized trials (RoB 2). Included studies: Li et al. [16], Ma et al. [17], Li et al. [18], Zhao et al. [19], Qu et al. [20], Min et al. [29], and Wei et al. [30].

Four trials showed an overall low risk of bias [16-18,30], two trials showed some concerns of bias [19,29], and another trial had an overall high risk of bias (Figure 2) [20]. Min et al. raised concerns about attrition bias, as 18 of 150 patients were lost to follow-up, and the reasons for this were not detailed [29]. Additionally, Y. Zhao et al. raised concerns about attrition bias, as they employed a per-protocol analysis [19]. Finally, Qu et al. showed a high risk of bias due to deviations from intended interventions because of the open-label study design [20]. Furthermore, A GRADE summary of evidence in Table 3 outlines the certainty of evidence.

**Table 3:** GRADE evidence profile GRADE: Grading of Recommendations Assessment, Development, and Evaluation; CI: confidence interval; MD: mean difference; RR: risk ratio; SMD: standardized mean difference. Explanations: a. Min et al. [29], Zhao et al. [19], and Qu et al. [20] showed some concerns or high risk of bias; b. I² > 50%; c. I² > 75%; d. A wide confidence interval that does not exclude appreciable harm or benefit; e. A low number of participants.

Certainty assessment							Summary of findings				
Participants (studies) Follow-up	Risk of bias	Inconsistency	Indirectness	Imprecision	Publication bias	Overall certainty of evidence	Study event rates (%)		Relative effect (95% CI)	Anticipated absolute effects	
							With (Control)	With (Esketamine)		Risk with (Control)	Risk difference with (Esketamine)
Pain at Rest, 12 hours											
577 (3 RCTs)	Serious^a^	Not serious	Not serious	Not serious	None	⨁⨁⨁◯ Moderate^a^	-	-	-	-	SMD 0.05 SD lower (0.21 lower to 0.12 higher)
Pain at Rest, 24 hours											
1009 (5 RCTs)	Serious^a^	Serious^b^	Not serious	Not serious	None	⨁⨁◯◯ Low^a,b^	-	-	-	-	SMD 0.2 SD lower (0.47 lower to 0.06 higher)
Pain during Activity, 12h											
577 (3 RCTs)	Serious^a^	Not serious	Not serious	Not serious	None	⨁⨁⨁◯ Moderate^a^	-	-	-	-	SMD 0.28 SD lower (0.45 lower to 0.12 lower)
Pain during Activity, 24h											
1009 (5 RCTs)	Serious^a^	Very serious^c^	Not serious	Serious^d^	None	⨁◯◯◯ Very low^a,c,d^	-	-	-	-	SMD 0.28 SD lower (0.56 lower to 0.01 higher)
Surgery Duration											
1101 (7 RCTs)	Not serious	Not serious	Not serious	Serious^d^	None	⨁⨁⨁◯ Moderate^d^	-	-	-	-	MD 2.06 minutes lower (4.02 lower to 0.11 lower)
Propofol Consumption											
444 (3 RCTs)	Serious^a^	Not serious	Not serious	Not serious	None	⨁⨁⨁◯ Moderate^a^	-	-	-	-	SMD 0.04 SD lower (0.22 lower to 0.15 higher)
Length of Hospital Stay											
649 (4 RCTs)	Not serious	Not serious	Not serious	Not serious	None	⨁⨁⨁⨁ High	-	-	-	-	MD 0.02 days lower (0.28 lower to 0.23 higher)
Depression at POD 1											
281 (3 RCTs)	Serious^a^	Serious^b^	Not serious	Serious^e^	None	⨁◯◯◯ Very low^a,b,e^	-	-	-	-	SMD 0.12 SD lower (0.47 lower to 0.22 higher)
Depression at POD 7											
334 (3 RCTs)	Very serious^a^	Very serious^c^	Not serious	Very serious^d,e^	None	⨁◯◯◯ Very low^a,c,d,e^	-	-	-	-	SMD 0.47 SD lower (1 lower to 0.06 higher)
Anxiety at POD 1											
280 (3 RCTs)	Very serious^a^	Very serious^c^	Not serious	Very serious^d,e^	None	⨁◯◯◯ Very low^a,c,d,e^	-	-	-	-	SMD 0.24 SD lower (0.72 lower to 0.25 higher)
Anxiety at POD 7											
335 (3 RCTs)	Very serious^a^	Very serious^c^	Not serious	Very serious^d,e^	None	⨁◯◯◯ Very low^a,c,d,e^	-	-	-	-	SMD 0.72 SD lower (1.4 lower to 0.04 lower)
Delirium											
808 (4 RCTs)	Serious^a^	Not serious	Not serious	Serious^d^	None	⨁⨁◯◯ Low^a,d^	31/403 (7.7%)	27/405 (6.7%)	RR 0.85 (0.52 to 1.40)	31/403 (7.7%)	12 fewer per 1,000 (from 37 fewer to 31 more)
Hallucinations											
785 (4 RCTs)	Not serious	Not serious	Not serious	Not serious	None	⨁⨁⨁⨁ High	3/393 (0.8%)	17/392 (4.3%)	RR 4.36 (1.48 to 12.88)	3/393 (0.8%)	26 more per 1,000 (from 4 more to 91 more)
Nightmares											
857 (5 RCTs)	Not serious	Not serious	Not serious	Not serious	None	⨁⨁⨁⨁ High	11/427 (2.6%)	13/430 (3.0%)	RR 1.20 (0.58 to 2.46)	11/427 (2.6%)	5 more per 1,000 (from 11 fewer to 38 more)
PONV											
1101 (7 RCTs)	Serious^a^	Not serious	Not serious	Not serious	None	⨁⨁⨁◯ Moderate^a^	165/547 (30.2%)	148/554 (26.7%)	RR 0.88 (0.73 to 1.07)	165/547 (30.2%)	36 fewer per 1,000 (from 81 fewer to 21 more)

Primary Outcome: Pain

**Figure 3:**
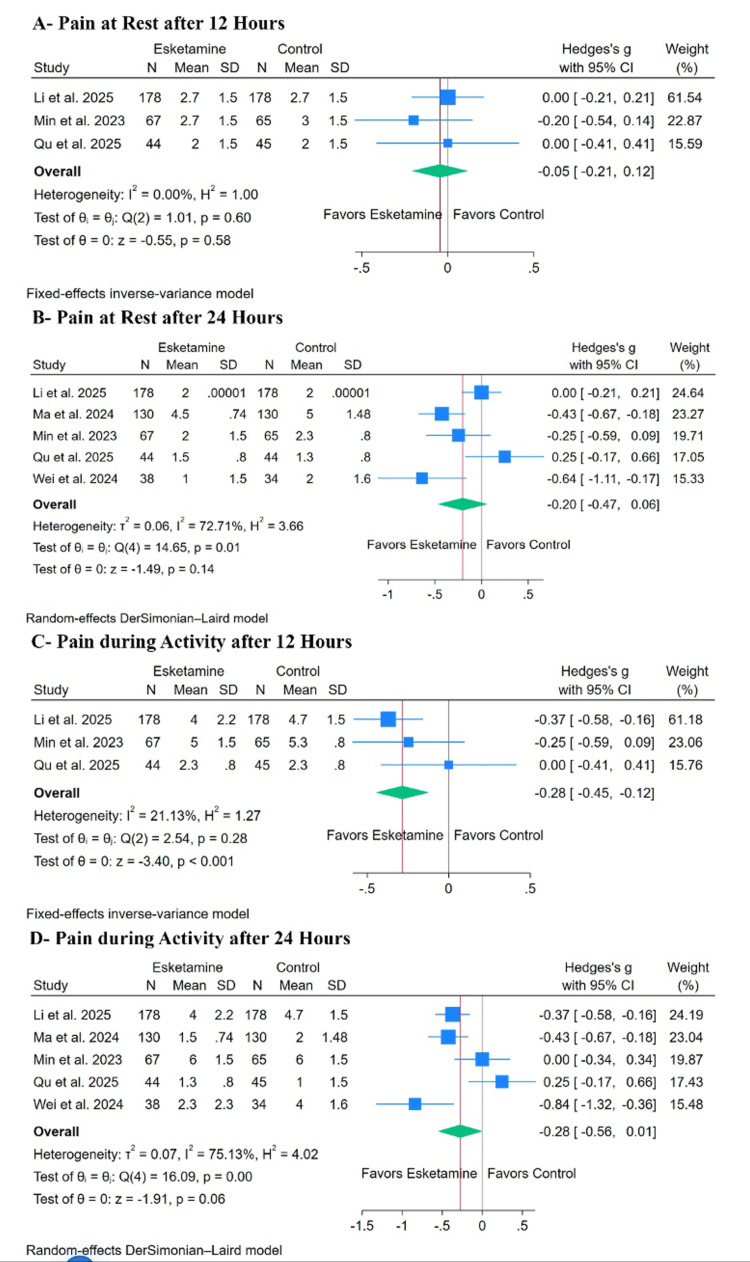
Forest plots of the primary outcome (pain). Forest plots demonstrating the effect of esketamine versus control on postoperative pain outcomes. Outcomes include pain at rest and pain during activity at various postoperative time points. Effect sizes are presented as standardized mean differences (SMDs) with 95% confidence intervals (CI). Included studies: Li et al. [16], Ma et al. [17], Li et al. [18], Zhao et al. [19], Qu et al. [20], Min et al. [29], and Wei et al. [30].

Esketamine did not significantly decrease pain at rest after 12 hours (SMD: -0.05, with 95% CI (-0.21, 0.12), p= 0.58, I²= 0%) (Figure 3A) or after 24 hours (SMD: -0.20, with 95% CI (-0.47, 0.06), p= 0.14, I²= 72.71%) (Figure 3B). However, esketamine significantly decreased pain during activity after 12 hours (SMD: -0.28, with 95% CI (-0.45, -0.12), p < 0.001, I²= 21.13%) (Figure 3C) but not after 24 hours (SMD: -0.28, with 95% CI (-0.56, 0.01), p= 0.06, I²= 75.13%) (Figure 3D).

For pain at rest after 24 hours, leave-one-out sensitivity analysis demonstrated instability of the pooled estimate, as the statistical significance of the result changed after omission of the Qu et al. [20] study. This finding suggests that the pooled effect may be sensitive to individual study influence and should therefore be interpreted cautiously (Appendix C, pane A). The Galbraith plot suggested that Qu et al. and Ma et al. were potential sources of the observed heterogeneity (Appendix C, pane B). For pain during activity after 24 hours, leave-one-out sensitivity analysis similarly demonstrated fragility of the pooled estimate, as statistical significance emerged only after exclusion of individual studies (Min et al. [29] or Qu et al. [20]). This suggests limited robustness of the observed effect (Appendix D, pane A). The corresponding Galbraith plot showed that Qu et al. are potential outliers and may be responsible for the observed heterogeneity (Appendix D, pane B).

Finally, the test for subgroup difference was not significant for pain at rest after 12 hours, either based on surgery type or timing of administration (P > 0.1) (Appendix E). However, significant pain at rest was observed after 24 hours, regardless of surgery type or timing of administration (P = 0.03) (Appendix F). Finally, it was not significant during activity in all scenarios (P > 0.1) (Appendices G, H).

Secondary Outcomes. 

**Figure 4:**
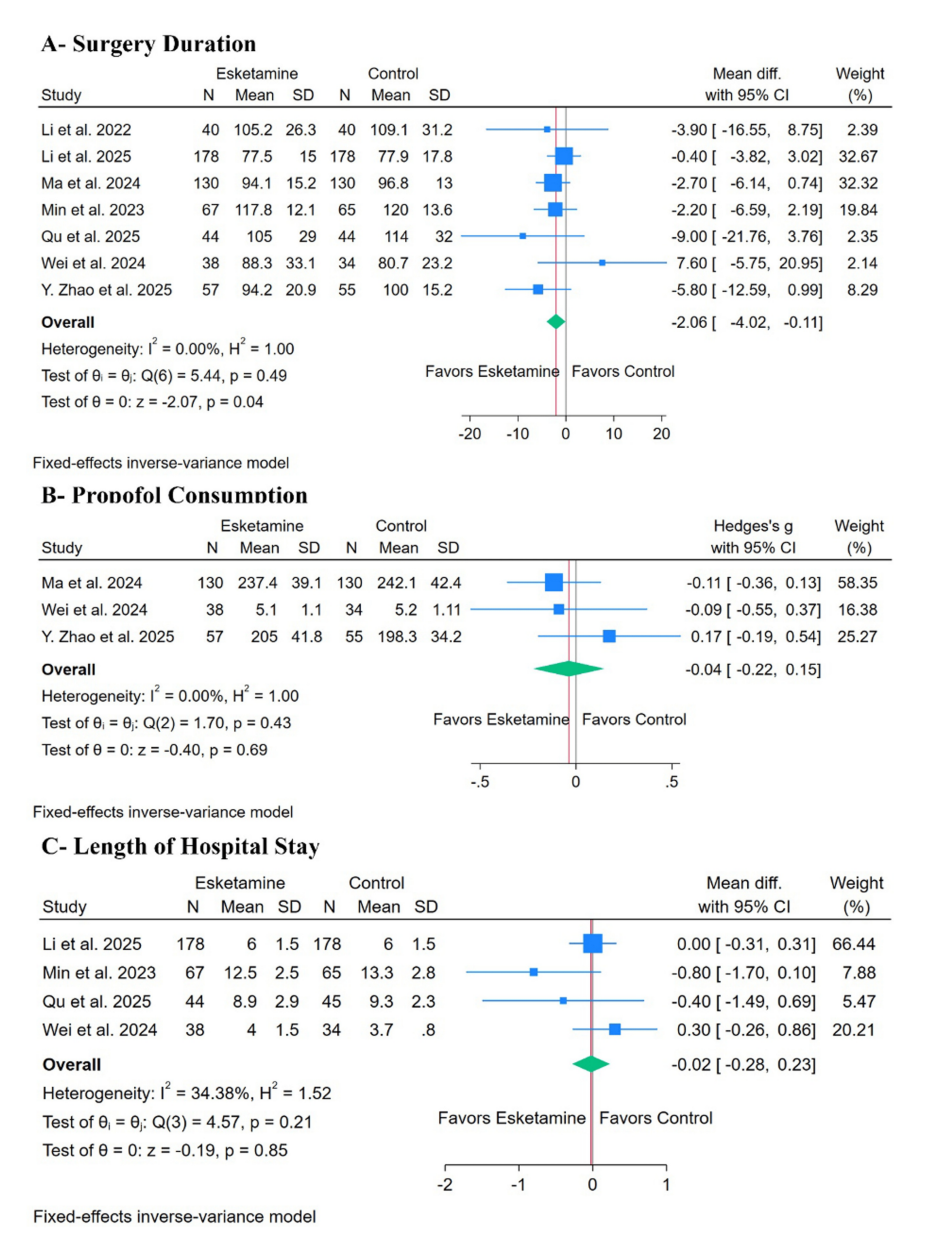
Forest plots of the secondary intra-operative outcomes. Forest plots demonstrating the effect of esketamine versus control on intra-operative and peri-operative outcomes. Outcomes include duration of surgery, peri-operative propofol consumption, and length of hospital stay. Effect sizes are presented as standardized mean differences (SMDs) with 95% confidence intervals (CI). Included studies: Li et al. [16], Ma et al. [17], Li et al. [18], Zhao et al. [19], Qu et al. [20], Min et al. [29], and Wei et al. [30].

Intra-operative efficacy outcomes: Esketamine significantly decreased the surgery duration (MD: -2.06 minutes, with 95% CI (-4.02, -0.11), p= 0.04, I²= 0%) (Figure 4A). However, there was no significant difference between both groups in propofol consumption (SMD: -0.04, with 95% CI (-0.22, 0.15), p= 0.69, I²= 0%) (Figure 4B). The test for subgroup difference was not significant for surgery duration, either based on surgery type or timing of administration (P > 0.1) (Appendices I, J). 

Post-operative efficacy outcomes: There was no significant difference between the two groups in the length of hospital stay (MD: -0.02 days, with 95% CI (-0.28, 0.23), p= 0.85, I²= 34.38%) (Figure 4C).

**Figure 5:**
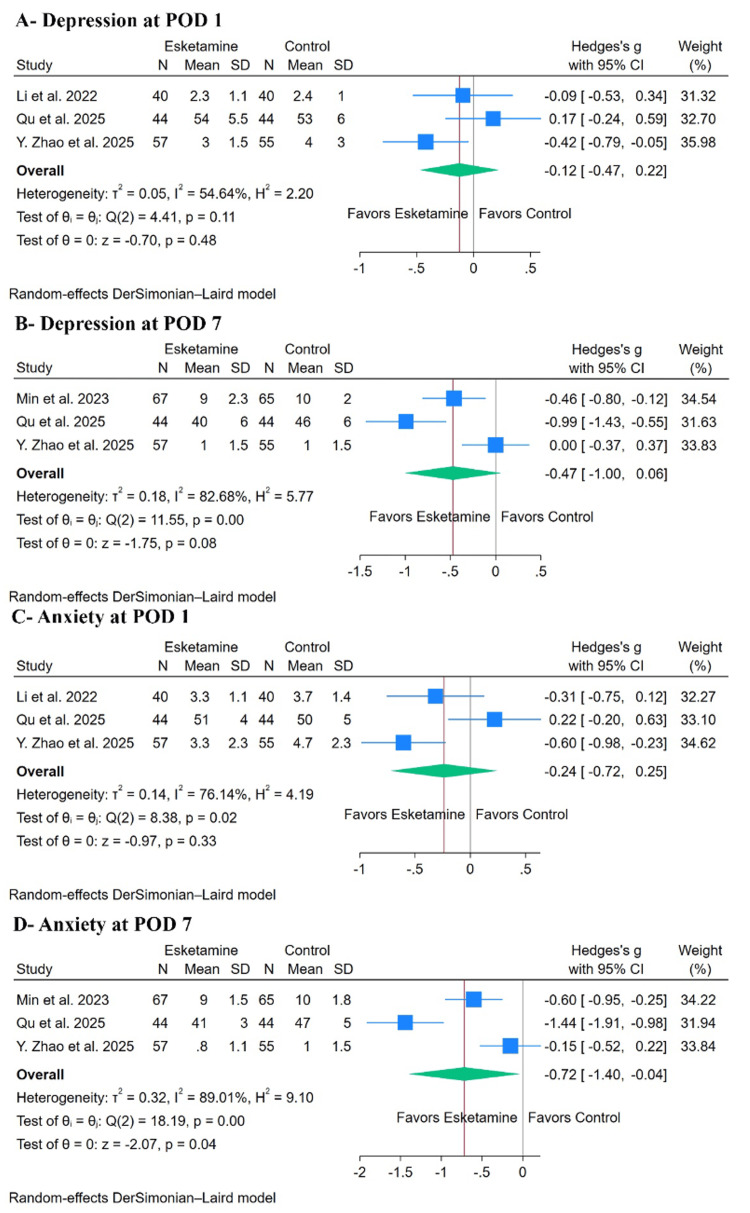
Forest plots of the secondary post-operative outcomes. Forest plots demonstrating the effect of esketamine versus control on postoperative psychological outcomes. Outcomes include postoperative anxiety and depression at various time points. Effect sizes are presented as standardized mean differences (SMDs/Hedges’ g) with 95% confidence intervals (CIs). Included studies: Li et al. [16], Ma et al. [17], Li et al. [18], Zhao et al. [19], Qu et al. [20], Min et al. [29], and Wei et al. [30]. POD: post-operative day

Regarding post-operative mood, esketamine did not significantly decrease depression scores at post-operative day 1 (SMD: -0.12, with 95% CI (-0.47, 0.22), p= 0.48, I²= 54.64%) (Figure 5A) or day 7 (SMD: -0.47, with 95% CI (-1.00, 0.06), p= 0.08, I²= 82.68%) (Figure 5B). Leave-one-out sensitivity analysis for depression at day 1 yielded consistent, non-significant results regardless of which study was omitted (Appendix K, pane A). The Galbraith plot indicated that Y. Zhao et al. were a potential source of heterogeneity (Appendix K, pane A). For depression at post-operative day 7, sensitivity analysis demonstrated instability of the pooled estimate, as statistical significance depended on exclusion of the Y. Zhao et al. study, indicating limited robustness of the finding (p = 0.007) (Appendix L, pane A), and the Galbraith plot identified Qu et al. as a potential outlier (Appendix L, pane B).

For anxiety, there was no difference between groups at post-operative day 1 (SMD: -0.24, with 95% CI (-0.72, 0.25), p= 0.33, I²= 76.14%) (Figure 5C). Sensitivity analysis for anxiety at post-operative day 1 demonstrated instability of the pooled estimate, as statistical significance emerged only after exclusion of the Qu et al. study (p = 0.001) (Appendix M, pane A), while the Galbraith plot identified Y. Zhao et al. as a source of heterogeneity (Appendix M, pane B). However, esketamine significantly decreased anxiety scores at post-operative day 7 (SMD: -0.72, with 95% CI (-1.40, -0.04), p = 0.04, I² = 89.01%) (Figure 5D). Leave-one-out sensitivity analysis showed that the statistically significant reduction in anxiety at post-operative day 7 became non-significant after exclusion of the Qu et al. study, suggesting that this finding may not be robust and should be interpreted cautiously (p = 0.090) (Appendix N, pane A). The Galbraith plot for this outcome identified Qu et al. as a significant outlier and source of heterogeneity (Appendix M, pane B).

**Figure 6:**
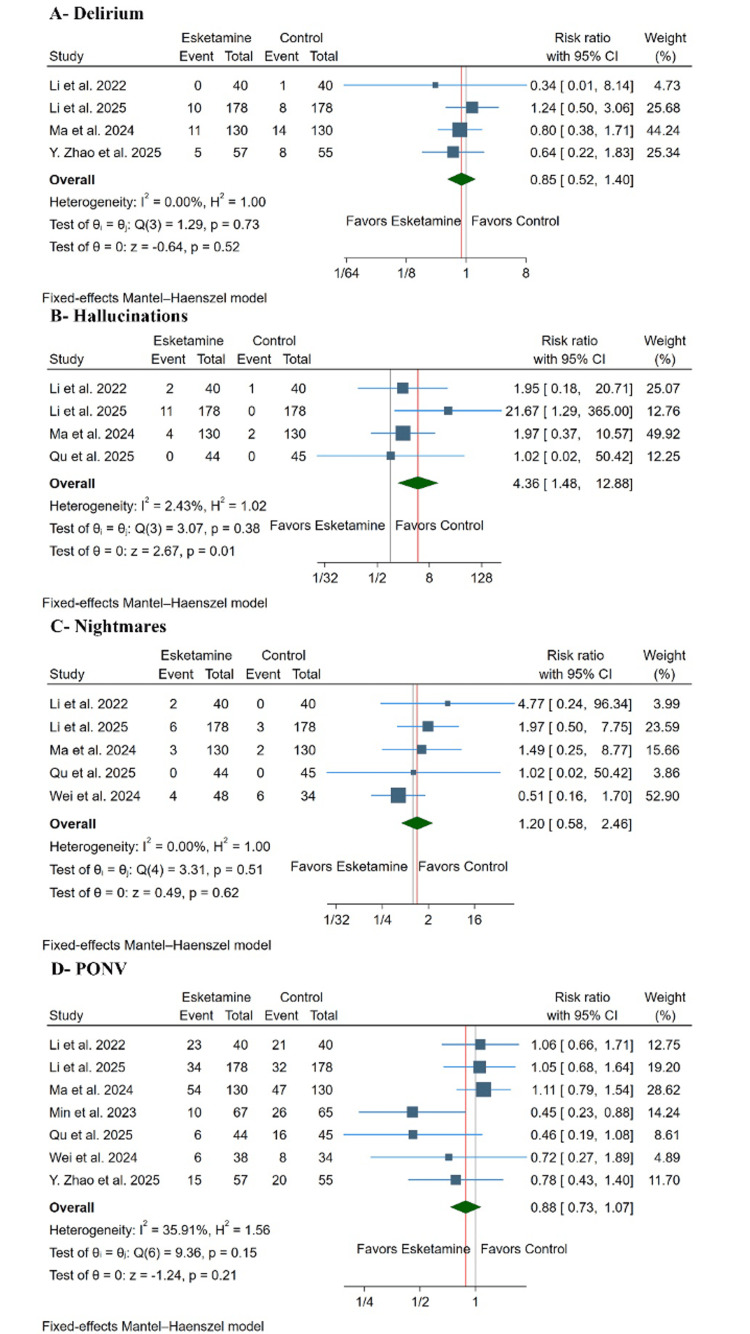
Forest plots of the safety outcomes. Forest plots demonstrating the effect of esketamine versus control on postoperative safety outcomes. Outcomes include hallucinations, postoperative nausea and vomiting (PONV), delirium, and nightmares. Effect sizes are presented as risk ratios (RRs) with 95% confidence intervals (CI). Included studies: Li et al. [16], Ma et al. [17], Li et al. [18], Zhao et al. [19], Qu et al. [20], Min et al. [29], and Wei et al. [30].

Safety outcomes: Esketamine significantly increased the risk of post-operative hallucinations (RR: 4.36, 95% CI (1.48, 12.88), p = 0.01) (Figure 6B). Across included studies, hallucinations occurred in approximately 4.3% of patients receiving esketamine compared with 0.8% in control groups, corresponding to an absolute risk increase of approximately 3.5% and an estimated number needed to harm (NNH) of 29. However, there was no significant difference between both groups in the incidence of delirium (RR: 0.85, with 95% CI (0.52, 1.40), p= 0.52, I²= 0%) (Figure 6A), nightmares (RR: 1.20, with 95% CI (0.58, 2.46), p= 0.62, I²= 0%) (Figure 6C), or PONV (RR: 0.88, with 95% CI (0.73, 1.07), p= 0.21, I²= 35.01%) (Figure 6D).

Discussion

This systematic review and meta-analysis synthesized data from seven RCTs encompassing 1,101 patients to evaluate the efficacy and safety of peri-operative esketamine in THA and TKA. The analysis showed a statistically significant but temporary decrease in post-operative pain, particularly during activity at 12 hours, along with a modest delayed anxiolytic effect observed seven days after surgery. However, the observed effect sizes were generally small (e.g., SMD: -0.28 for activity-related pain at 12 hours), suggesting limited clinical impact despite statistical significance. These limited benefits were contrasted with a statistically significant increase in hallucinations in the esketamine group. Still, the analysis demonstrated no significant advantage for several critical outcomes, including pain at rest, post-operative depression, post-operative delirium, and key recovery metrics, such as LOS. 

The short-term reduction in activity-related pain at 12 hours may reflect esketamine’s anti-hyperalgesic properties through non-competitive NMDA receptor antagonism [31,32]. By reducing central sensitization, esketamine may transiently improve post-operative pain during mobilization. However, this benefit appeared short-lived, as no significant improvement persisted at 24 hours. Given the small pooled effect size and temporary duration, the overall clinical relevance of this analgesic benefit remains uncertain.

The reduction in post-operative anxiety on post-operative day 7 is also of interest and may relate to esketamine’s known antidepressant and anxiolytic properties mediated through glutamatergic modulation and downstream neurotrophic signaling [33,34]. However, this finding demonstrated important instability during sensitivity analyses, as exclusion of the Qu et al. study [20] rendered the pooled result non-significant. Several statistically significant findings were similarly sensitive to exclusion of individual studies during leave-one-out analyses, indicating limited robustness of some pooled estimates.

Despite modest short-term improvements in pain and anxiety outcomes, these benefits were accompanied by a statistically significant increase in post-operative hallucinations. Although esketamine has been proposed to provide effective multimodal analgesia with fewer psychomimetic effects than racemic ketamine [35-37], our findings suggest that clinically relevant psychological adverse effects remain an important concern. These experiences may be particularly distressing in older arthroplasty patients and could negatively influence post-operative recovery and patient perception of surgical care.

Our analysis showed no significant increase in the risk of post-operative delirium or nightmares. The absence of any impact on delirium is interesting, considering ketamine's paradoxical history, as it has been studied as a possible preventive measure and identified as a potential contributor to delirium [35]. The difference between a notable rise in hallucinations and the absence of delirium might indicate that esketamine triggers a particular perceptual disturbance instead of a general cognitive issue defined by inattention and inconsistent awareness. The included trials did not explicitly investigate mitigation strategies of this effect, such as the co-administration of benzodiazepines [38], which are described in the literature. Such strategies could also introduce risks in elderly surgical patients, like over-sedation and potential delirium [37]. 

Furthermore, while our study focused on psychomimetic and delirium-related safety, the broader peri-operative management of THA and TKA involves vigilance against other critical complications, such as venous thromboembolism [39,40], periprosthetic infection [41], and adverse local reactions to implant materials [42]. Therefore, future studies may consider these complications while assessing esketamine safety.

Limitations

Several limitations should be considered when interpreting the findings of this meta-analysis. First, all included studies were conducted in China, which limits the generalizability and external applicability of the findings to other healthcare systems and patient populations. Differences in peri-operative protocols, multimodal analgesic strategies, healthcare delivery systems, and potentially cultural patterns of symptom reporting may influence both efficacy and safety outcomes across different regions.

Second, the included studies demonstrated substantial clinical heterogeneity regarding esketamine dosing regimens, timing of administration (intra-operative, post-operative, or both), peri-operative analgesic protocols, surgical populations (THA, TKA, or mixed cohorts), and outcome assessment methods. This variability limits the ability to attribute observed effects to a specific esketamine strategy and complicates straightforward clinical application.

Third, several statistically significant findings demonstrated instability during leave-one-out sensitivity analyses, suggesting limited robustness of some pooled estimates. In addition, the relatively small number of included studies for several pooled outcomes may reduce the precision and stability of the calculated effect estimates.

Fourth, publication bias and small-study effects should also be considered. Because fewer than 10 studies were available for most pooled outcomes, formal assessment using funnel plots or statistical asymmetry testing was not considered reliable. Consequently, positive findings may be overrepresented in the available literature, particularly for subjective outcomes such as pain and psychological measures.

Finally, although conventional random-effects meta-analysis using the DL estimator was applied, recent methodological literature suggests that this approach may underestimate uncertainty in the presence of substantial heterogeneity, potentially leading to narrower confidence intervals compared with more robust estimators such as Restricted Maximum Likelihood (REML) or Hartung-Knapp adjustment methods [43]. Therefore, statistically significant findings, particularly those with borderline p-values, should be interpreted cautiously.

Implications for Future Research

The current evidence base has considerable limitations, creating a clear and urgent need for future research. Future studies should prioritize a standard esketamine protocol to address the clinical variability that affected this analysis. Head-to-head comparisons of administration during surgery versus after surgery would be particularly valuable. Additionally, outcome assessment should not be limited to basic pain scores. Still, it should also incorporate validated, patient-centered measures, such as the Quality of Recovery-15 score, functional milestones, and long-term outcomes [44]. Safety monitoring requires enhancement; the proactive and systematic evaluation of psychomimetic effects using validated scales is necessary, rather than simply recording spontaneously reported adverse events. Finally, exploring strategies to reduce psychomimetic effects, such as co-administering dexmedetomidine, which may provide synergistic analgesia while opposing psychiatric symptoms, signifies an additional avenue for investigation. 

Implications for Clinical Practice

It is essential to interpret our findings within the broader context of peri-operative care. Post-operative recovery following THA and TKA is a multifactorial process driven primarily by the extent of surgical trauma, the efficacy of multimodal analgesia, rehabilitation protocols, and individual risk factors [45]. While esketamine possesses unique pharmacological properties that may mitigate central sensitization, it is short-acting [46]. Therefore, the physiological plausibility that a brief intra-operative or peri-operative exposure to esketamine can meaningfully alter long-term outcomes such as mood or functional recovery remains debatable. The limited benefits observed in this meta-analysis may reflect the reality that a single pharmacological intervention is insufficient to override the subtle physiological and psychological stressors associated with major joint replacement surgery.

Additionally, it is critical to interpret some of our statistically significant findings within the context of clinical significance. Although esketamine reduced surgery duration by a mean of 2.06 minutes (p = 0.04), this difference falls below the threshold of clinical meaningfulness for major joint arthroplasty. Similarly, regarding analgesia, established literature suggests that the minimal clinically important difference (MCID) for post-operative pain on the 100-point VAS is approximately 10 mm to 20 mm [47]. Still, our analysis showed an SMD of -0.28 for pain during movement, which generally translates to a raw reduction of less than 10 mm on the VAS, suggesting the analgesic benefit may be marginal or sub-clinical for many patients. In contrast, the reduction in anxiety on post-operative day 7 (SMD -0.72) represents a medium-to-large effect size that likely exceeds the MCID for hospital-associated anxiety [48], pointing to a potentially meaningful psychiatric benefit rather than an analgesic.

## Conclusions

In patients undergoing THA or TKA, peri-operative esketamine may provide a modest and short-term reduction in activity-related postoperative pain. A possible delayed reduction in postoperative anxiety was also observed; however, this finding demonstrated instability during sensitivity analyses and should be interpreted cautiously. Esketamine was additionally associated with a significantly increased risk of post-operative hallucinations, while no significant differences were observed in delirium, nightmares, or postoperative nausea and vomiting. Overall, the clinical benefits appear limited and temporary, and routine peri-operative use of esketamine in this setting cannot currently be strongly recommended given the imbalance between modest benefits and potential neuropsychiatric adverse effects.

## Figures and Tables

**Figure 7:**
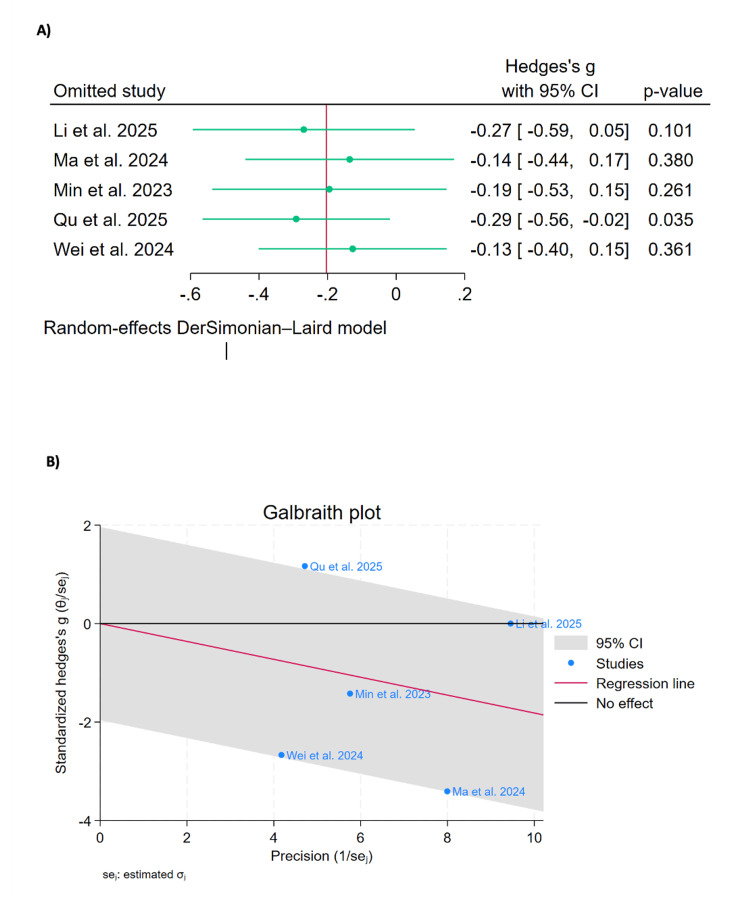
Sensitivity and heterogeneity analyses for pain at rest after 24 hours (A) Leave-one-out sensitivity analysis assessing the robustness of pooled pain-at-rest outcomes at 24 hours. (B) Galbraith plot evaluating heterogeneity among included studies for pain at rest after 24 hours.

**Figure 8:**
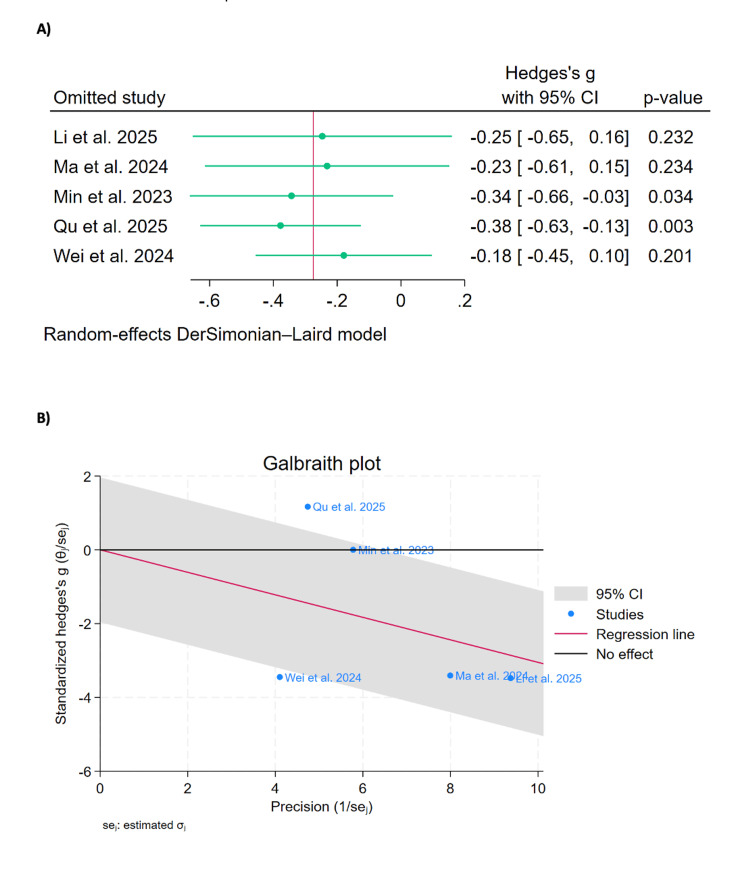
Sensitivity and heterogeneity analyses for pain during activity at 24 hours (A) Leave-one-out sensitivity analysis assessing the robustness of pooled pain-during-activity outcomes at 24 hours. (B) Galbraith plot evaluating heterogeneity among included studies for pain during activity at 24 hours.

**Figure 9:**
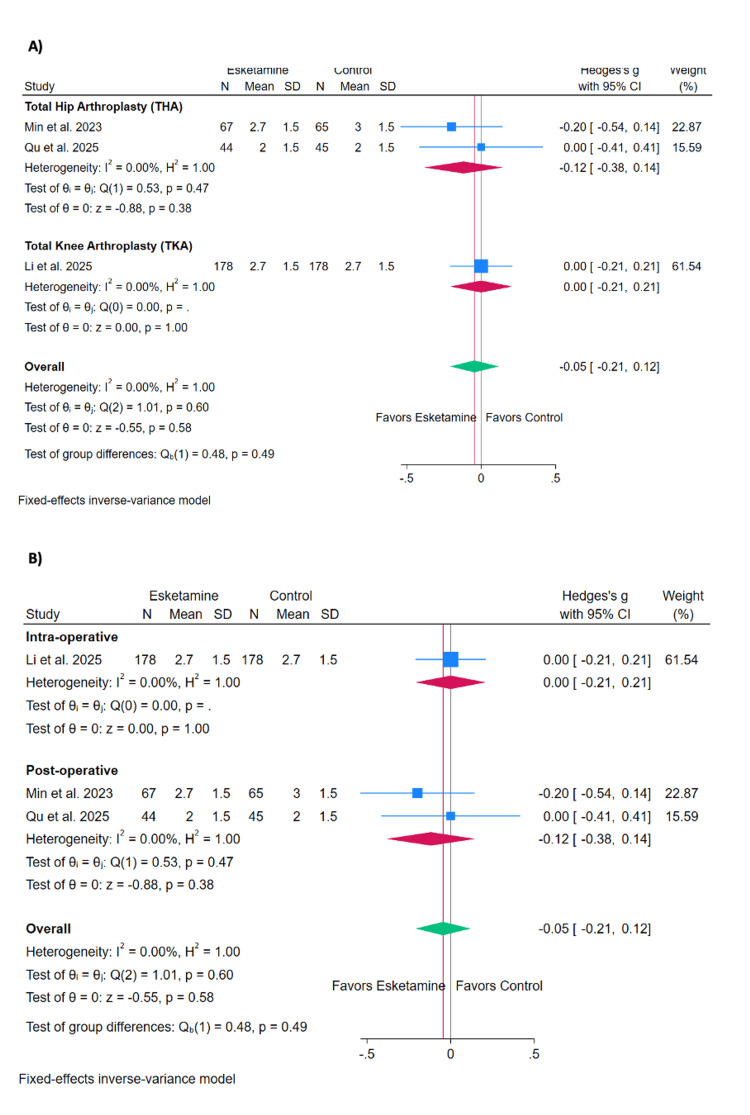
Subgroup analyses for pain at rest after 12 hours (A) Subgroup analysis based on surgery type for pain at rest after 12 hours. (B) Subgroup analysis based on timing of esketamine administration for pain at rest after 12 hours.

**Figure 10:**
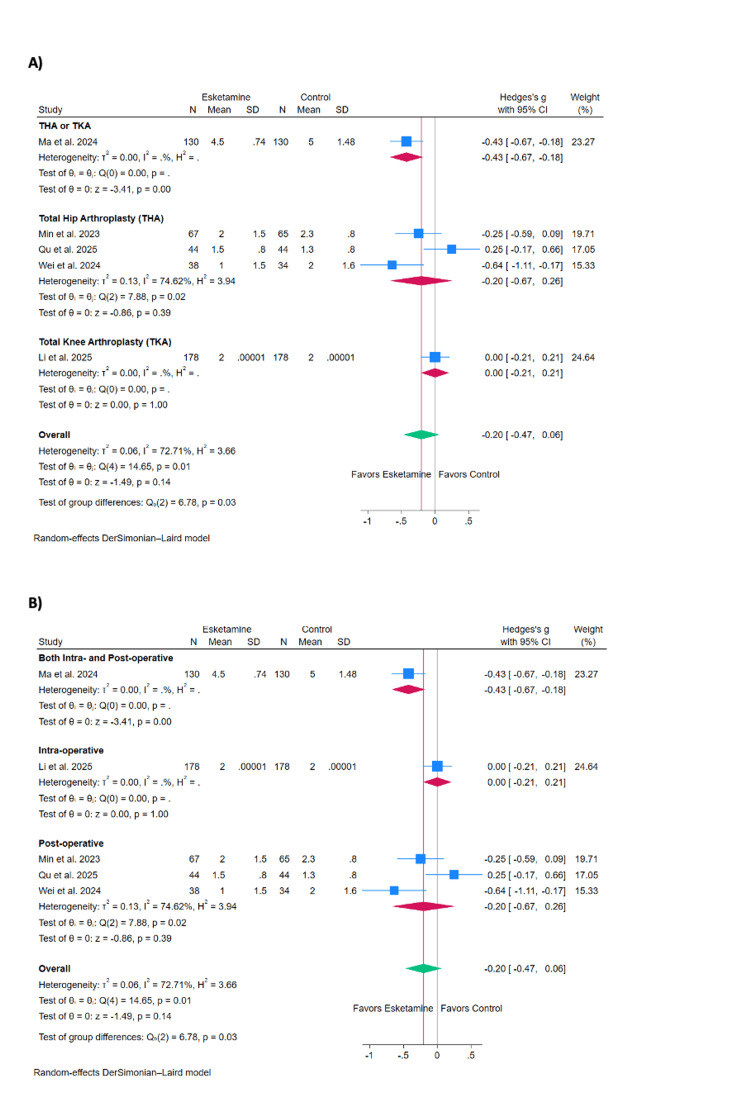
Subgroup analyses for pain at rest after 24 hours (A) Subgroup analysis based on surgery type for pain at rest after 24 hours. (B) Subgroup analysis based on timing of esketamine administration for pain at rest after 24 hours.

**Figure 11:**
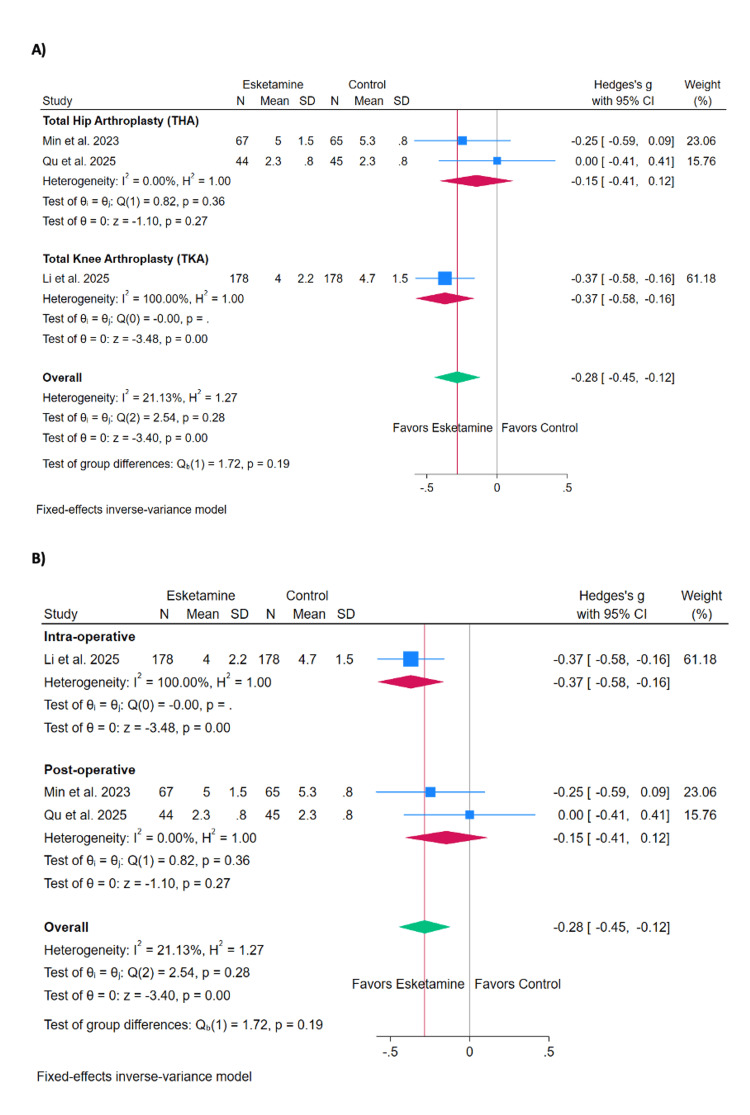
Subgroup analyses for pain during activity after 12 hours (A) Subgroup analysis based on surgery type for pain during activity after 12 hours. (B) Subgroup analysis based on timing of esketamine administration for pain during activity after 12 hours.

**Figure 12:**
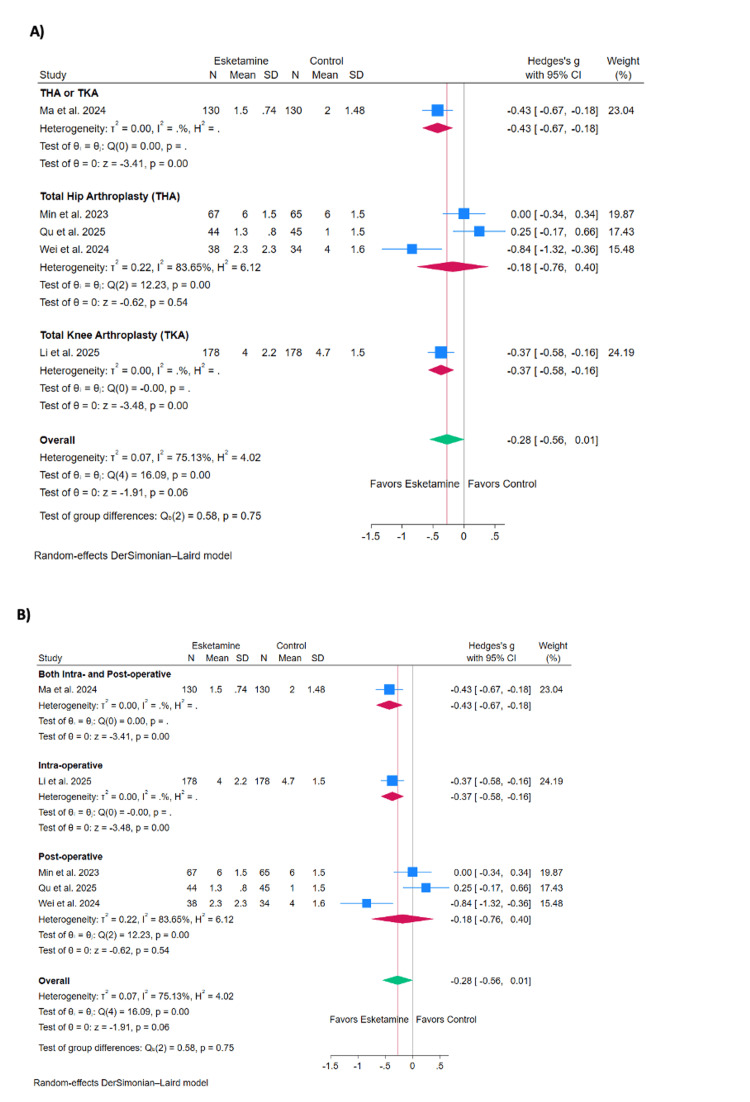
Subgroup analyses for pain during activity after 24 hours (A) Subgroup analysis based on surgery type for pain during activity after 24 hours. (B) Subgroup analysis based on timing of esketamine administration for pain during activity after 24 hours.

**Figure 13:**
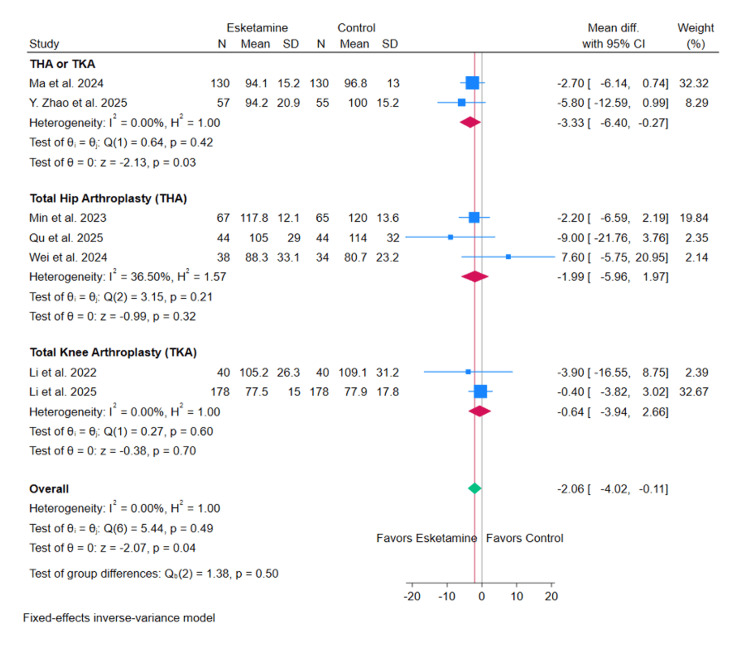
Subgroup analysis based on surgery type for surgery duration

**Figure 14:**
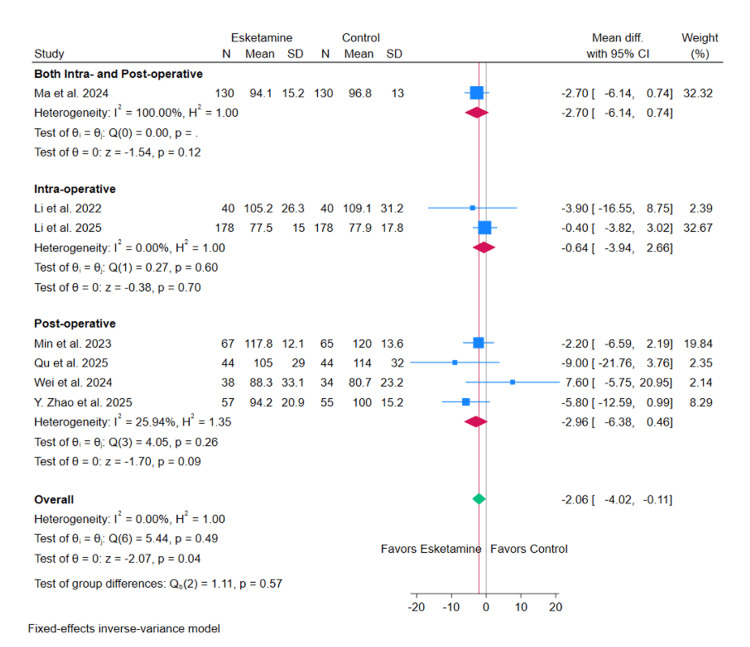
Subgroup analysis based on timing of esketamine administration for surgery duration

**Figure 15:**
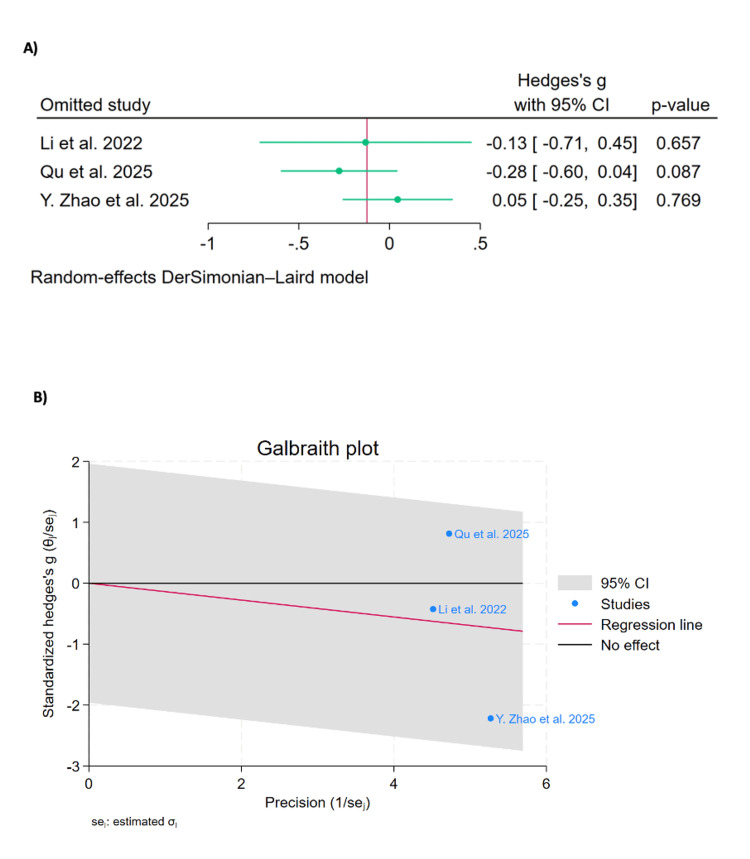
Sensitivity and Galbraith analyses for depression at postoperative day 1 (A) Leave-one-out sensitivity analysis of depression at postoperative day 1. (B) Galbraith plot of depression at postoperative day 1.

**Figure 16:**
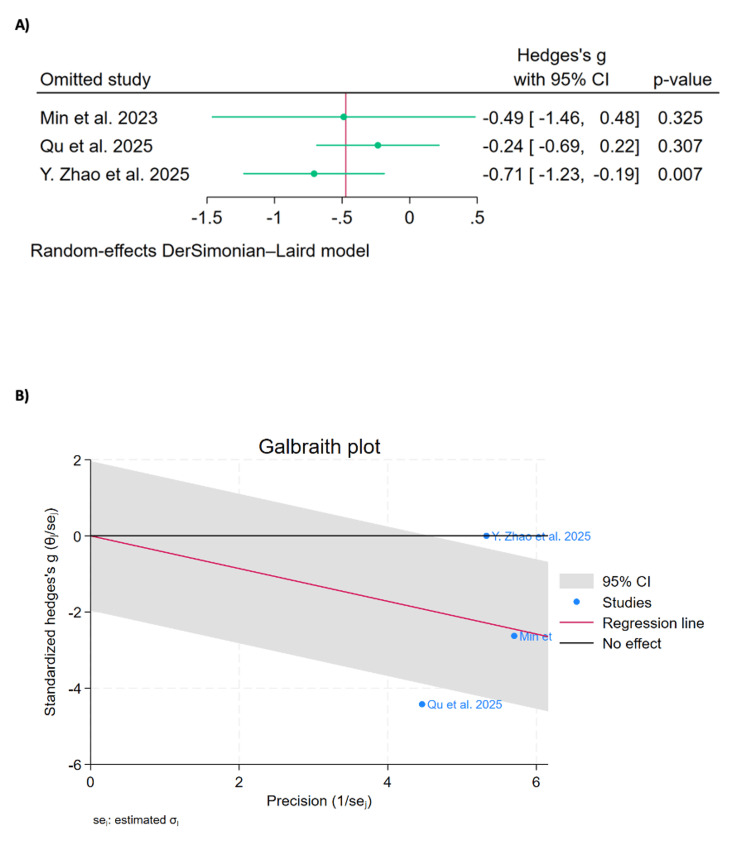
Sensitivity and Galbraith analyses for depression at postoperative day 7 (A) Leave-one-out sensitivity analysis of depression at postoperative day 7. (B) Galbraith plot of depression at postoperative day 7.

**Figure 17:**
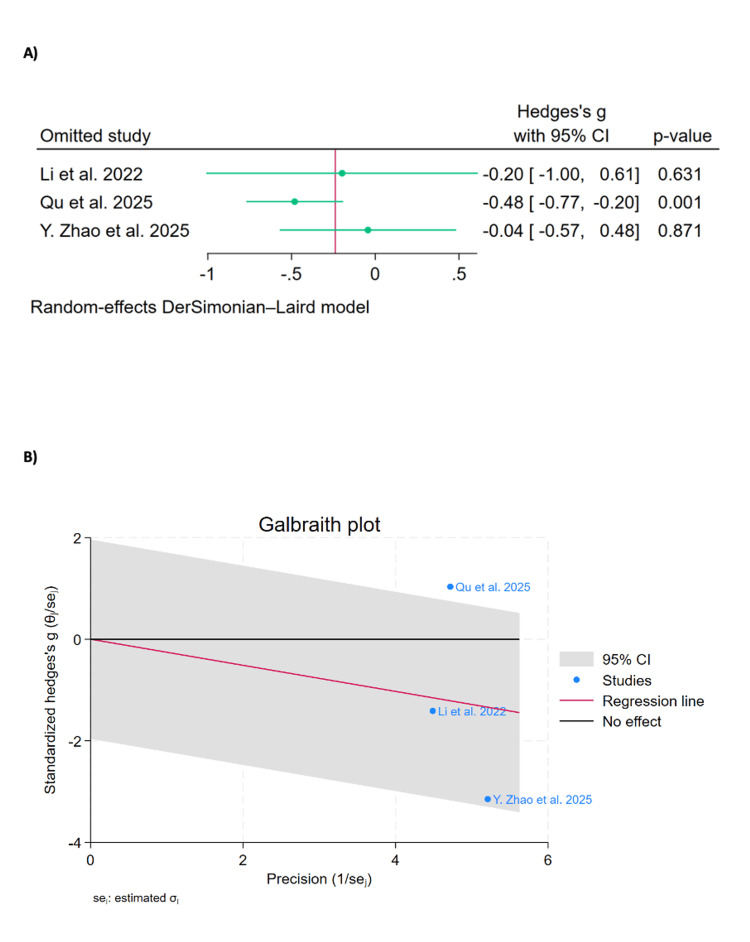
Sensitivity and Galbraith analyses for anxiety at postoperative day 1 (A) Leave-one-out sensitivity analysis of anxiety at postoperative day 1. (B) Galbraith plot of anxiety at postoperative day 1.

**Figure 18:**
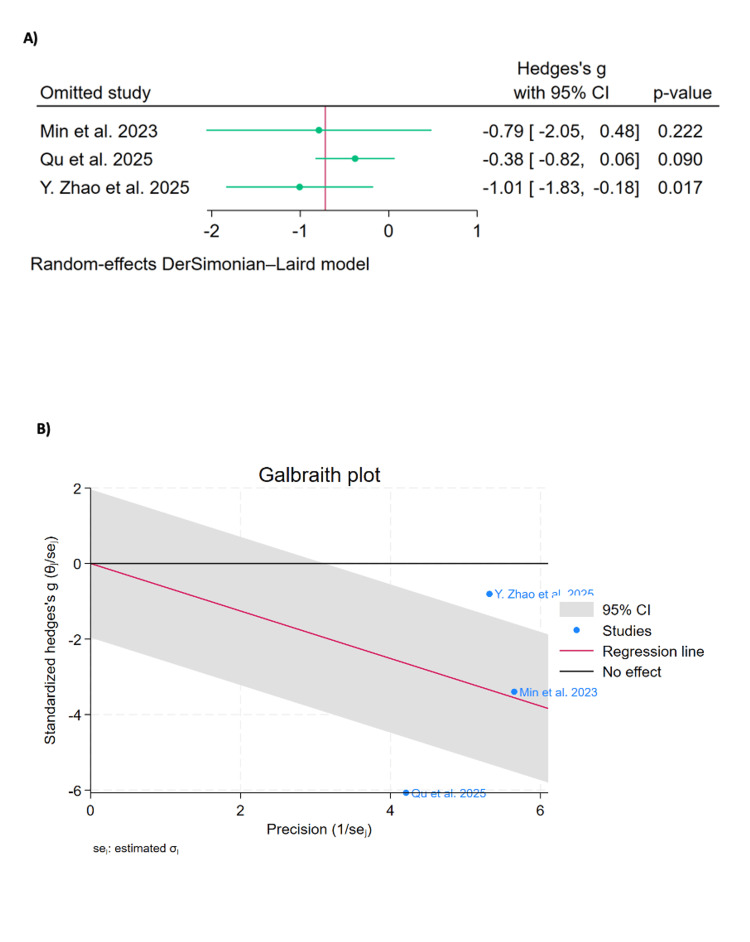
Sensitivity and Galbraith analyses for anxiety at postoperative day 7 (A) Leave-one-out sensitivity analysis of anxiety at postoperative day 7. (B) Galbraith plot of anxiety at postoperative day 7.

## Appendices

Appendix A 

**Table 4:** Search strategy

Database	Search Terms	Search Field	Search Results
Pubmed	("Esketamine" OR "S-ketamine” OR "S ketamine" OR "Spravato" OR "JNJ-54135419") AND (knee OR hip) AND (arthroplasty OR replac* OR prosthesis OR implant*)	All Fields	22
Cochrane	("Esketamine" OR "S-ketamine” OR "S ketamine" OR "Spravato" OR "JNJ-54135419") AND (knee OR hip) AND (arthroplasty OR replac* OR prosthesis OR implant*)	All Text	66
WOS	("Esketamine" OR "S-ketamine” OR "S ketamine" OR "Spravato" OR "JNJ-54135419") AND (knee OR hip) AND (arthroplasty OR replac* OR prosthesis OR implant*)	All Fields	26
SCOPUS	TITLE-ABS-KEY ( ( "Esketamine" OR "S-ketamine" OR "S ketamine" OR "Spravato" OR "JNJ-54135419" ) AND ( knee OR hip ) AND ( arthroplasty OR replac* OR prosthesis OR implant* ) )	Title, Abstract, keywords	38

Appendix B  

**Table 5:** Excluded records in full text screening

Title	Published Year	DOI	Study ID	Exclusion Reason
Effects of mini-dose esketamine-dexmedetomidine combination supplemented intravenous analgesia on postoperative sleep quality in elderly patients after total knee arthroplasty: a protocol of double-blind randomised trial.	2025	10.1136/bmjopen-2024-098018	Wang 2025	Study Protocol
Effects of S-ketamine on recovery quality in elderly patients with impaired intrinsic capacity after total knee arthroplasty: a single-centre, randomised, double-blind, placebo-controlled study protocol.	2025	10.1136/bmjopen-2024-094060	Liu 2025	Study Protocol
Effect of subanesthetic dose of esketamine on early postoperative depression in elderly patients with Sarcopenia.	2024	10.1186/s13018-024-05388-2	Fang 2024	Wrong study design
Evaluating the effects of S-ketamine on postoperative delirium in elderly patients following total hip or knee arthroplasty under intraspinal anesthesia: a single-center randomized, double-blind, placebo-controlled, pragmatic study protocol.	2023	10.3389/fnagi.2023.1298661	Zhu 2023	Study Protocol
Analgesic Effect of Intraoperative Intravenous S(+)-Ketamine During Total Knee Arthroplasty Surgery: Study Protocol for a Randomized Controlled Clinical Trial.	2023	10.2196/53063	Deng 2023	Study Protocol
A subanesthetic dose of esketamine combined with hip peripheral nerve block has good sedative and analgesic effects in elderly patients undergoing total hip arthroplasty: A randomized-controlled trial.	2023	10.52312/jdrs.2023.997	Dai 2023	Wrong intervention
Effects of esketamine on postoperative rebound pain in patients undergoing unilateral total knee arthroplasty: a single-center, randomized, double-blind, placebo-controlled trial protocol.	2023	10.3389/fneur.2023.1179673	Zhu 2023	Study Protocol
The effect of pregabalin and s-ketamine in total knee arthroplasty patients: A randomized trial.	2016	10.4103/0970-9185.194762	Kadic 2016	Wrong intervention
The effect of addition of pregabalin and s-ketamine to local infiltration analgesia on the knee function outcome after total knee arthroplasty.	2012	NA	Kadic 2012	Wrong intervention
Analgesic efficacy of the intra-articular administration of S(+)- ketamine in patients undergoing total knee arthroplasty.	2012	10.1016/S0034-7094(12)70165-4	GuarÃ¡Sobrinho 2012	Wrong intervention
Small-dose S(+)-ketamine reduces postoperative pain when applied with ropivacaine in epidural anesthesia for total knee arthroplasty.	2001	10.1097/00000539-200105000-00040	Himmelseher 2001	Wrong intervention
The Effect of Esketamine as an Adjuvant for Adductor Canal Block on Postoperative Pain in Patients Undergoing Arthroscopic Knee Surgery: A Randomized Controlled Trial	2025	10.62713/aic.3775	Zhao 2025	Wrong intervention
Analysis of Clinical Effect of Subanesthetic Dose of Esketamine Combined With Hip Capsule Peripheral Nerve Block in Elderly Patients Undergoing Total Hip Arthroplasty	2022	NCT05602428 2022	NCT05602428 2022	Study Protocol
Esketamine vs. Ketorolac for Prevention of Postoperative Pain and Cognitive Dysfunction After Total Knee Arthroplasty	2021	NCT05132595 2021	NCT05132595 2021	Study Protocol
Effect of esketamine combined with sufentanil on postoperative analgesic efficacy and cognitive function in elderly total knee replacement patients: a randomized, controlled, double-blind pilot study	2025	ChiCTR2500101951 2025	ChiCTR2500101951 2025	Study Protocol

Appendix C  

Appendix D  

Appendix E  

Appendix F  

Appendix G  

Appendix H  

Appendix I  

Appendix J 

Appendix K  

Appendix L  

Appendix M 

Appendix N  
